# Association Between Regular Physical Activity and Food-Specific Inhibitory Control in Young Chinese Adults: An fMRI Study

**DOI:** 10.3390/nu18030486

**Published:** 2026-02-01

**Authors:** Yali Liu, Jialong Zou, Zihan Sun, Yuting Zhang, Xiaokai Li, Peijie Chen

**Affiliations:** 1 School of Exercise and Health, Shanghai University of Sport, Shanghai 200438, China; 2311516020@sus.edu.cn (Y.L.);; 2 Magnetic Resonance Imaging Center, Shanghai University of Sport, Shanghai 200438, China

**Keywords:** regular physical activity, food-specific inhibitory control, stop-signal task, fMRI, healthy adults

## Abstract

**Background/Objectives**: Physical activity (PA) has been associated with better inhibitory control (IC), which may support self-regulatory processes related to eating. However, whether regular PA is related to food-specific IC and its neural correlates remains insufficiently understood. This cross-sectional study aimed to examine the relationship between regular PA, behavioral performance, and neural correlates of IC, with a focus on high-reward food-related contexts. **Methods**: Sixty-one healthy right-handed young Chinese adults were classified into a regular physical activity group (RPG; *n* = 30, 24 males) or an inactive group (IAG; *n* = 31, 17 males) based on self-reported frequency and volume of PA. Stop-signal tasks performed during functional MRI under high-calorie food and neutral image conditions were used to assess IC. Stop-signal reaction time (SSRT) indexed IC performance. Neural correlates of IC were examined using whole-brain and region-of-interest analyses, with brain activation values derived from general linear models including age, sex, body mass index, depressive scores, and subjective appetite ratings as covariates. Given the relatively small sample size and unbalanced distribution of sex and body mass index, sensitivity analyses were performed by varying covariate adjustments to assess the robustness of the primary results. **Results**: RPG demonstrated significantly shorter SSRT than IAG across both high-calorie food and neutral stimulus conditions. In contrast to successful-stop trials relative to baseline, IAG showed lower activation in the bilateral precuneus than RPG under the high-calorie food condition. In comparison, RPG showed lower activation than IAG under the neutral condition. In contrast to failed-stop trials relative to successful-go trials, IAG exhibited greater activation in the left caudate than RPG under the high-calorie food condition. These behavioral and neural patterns were generally robust across sensitivity analyses. **Conclusions**: Regular PA was associated with superior general IC, and this advantage was maintained in the presence of high-calorie food cues. At the neural level, regular PA was associated with stimulus-dependent neural responses in the bilateral precuneus and left caudate. Future studies using larger, more representative samples, objective measures of PA, and stratification by sex or BMI are warranted.

## 1. Introduction

Physical activity (PA) has been positively linked to general inhibitory control (IC) [1], a core executive function that enables individuals to maintain goal-directed behavior by suppressing irrelevant or distracting stimuli and inhibiting prepotent responses [2]. An efficient IC plays a critical role in self-regulation, such as diet. Neuroimaging evidence demonstrates that neural responses differ when individuals resist high-calorie food cues compared to neutral stimuli [3]. However, whether PA is differentially associated with general versus food-specific IC, at both the behavioral level and the level of underlying neural correlates, remains unclear.

IC is commonly assessed using well-established paradigms, including the stop-signal task (SST), go/no-go, Stroop, and flanker tasks [2,4]. Tasks incorporating hyperpalatable, calorie-dense food stimuli are commonly employed to assess food-specific IC, whereas tasks utilizing neutral stimuli are used to evaluate general IC. At the neural level, IC is primarily supported by frontal–parietal and subcortical regions, including the dorsolateral prefrontal cortex, inferior frontal gyrus, pre-supplementary motor area, and basal ganglia [5,6]. Furthermore, efficient IC relies on dynamic integration and interaction among large-scale brain networks, such as the frontoparietal network and the default mode network (DMN), across different task contexts [6]. Dysregulated activity in reward and executive control networks has been associated with maladaptive eating behaviors in environments rich in high-calorie food cues [7,8].

PA, defined as any bodily movement generated by skeletal muscles that results in energy expenditure [9], is commonly recognized as an important behavioral factor related to body composition. Individuals with chronic higher levels of PA often exhibit lower adiposity or a healthier body composition; however, this association is influenced by multiple covarying factors, including sex, age, metabolic profile, and psychological characteristics [10]. Beyond metabolic effects, a growing body of evidence suggests that higher levels of PA are associated with enhanced general IC and altered neural processes supporting cognitive control [11,12]. Improved general IC has been proposed as one pathway through which PA may support weight-related self-regulation [13]. Existing evidence further suggested PA has been linked to altered food reward processing and food preferences [14,15]. Nevertheless, whether regular PA is similarly associated with food-specific IC, and whether the underlying neural mechanisms differ from those supporting general IC, remains poorly understood.

Several studies have suggested that acute PA can transiently enhance food-specific IC by modulating electrophysiological indices of attentional control [16,17,18,19,20]. For example, Zhang et al. [16] reported that a 20-min session of moderate-intensity rope skipping significantly reduced reaction time (RT) in a food-word Stroop task among obese children. Similarly, Xie et al. [20] found that 20 min of high-intensity interval cycling reduced RT and increased the amplitudes of the P3 and late positive potential during a food-related flanker task in obese young males compared with a resting condition. Collectively, these findings indicate that acute PA may temporarily facilitate food-related IC. However, it remains unclear whether regular PA is associated with sustained differences in food-specific IC performance and how such differences are reflected at the neural level.

Functional MRI (fMRI) offers a valuable tool for exploring the neural correlates of PA-related effects on cognitive processing [21,22]. To date, most fMRI studies have focused on the impact of regular PA on general IC [23,24], demonstrating PA-related alterations in activation patterns across brain networks supporting cognitive control, including the DMN, executive control networks, and salience network [1,25]. For instance, Davis et al. [23] demonstrated that regular aerobic exercise improved cognitive performance, increased neural activation of bilateral prefrontal regions, and reduced activation in bilateral parietal areas of sedentary children. Similarly, Krafft et al. [24] reported regular aerobic exercise increased activation in the superior frontal gyrus and anterior cingulate cortex during a flanker task, while reducing activation in the precentral gyrus and posterior parietal cortex during an anti-saccade task. A recent activation likelihood estimation meta-analysis further indicated that exercise interventions significantly alter brain activation patterns during general cognitive tasks, particularly in frontal, precuneus, thalamic, and cingulate regions [25]. Nevertheless, the neural substrates of food-specific IC in the context of regular PA remain insufficiently investigated.

Collectively, these considerations highlight the importance of examining both behavioral and neural correlates of food-specific IC in individuals with differing levels of PA. To address this, the present study employed the SST during fMRI to examine IC performance and associated brain activation patterns. Stop-signal reaction time (SSRT), derived from the horse-race model, served as an objective measure of IC [26,27]. It was hypothesized that individuals engaging in regular PA would exhibit shorter SSRTs and stimulus-dependent neural activation patterns in regions supporting IC. Although causal relationships cannot be inferred, these findings provide a basis for future research investigating how PA and cognitive control interact in the context of dietary decision-making.

## 2. Materials and Methods

### 2.1. Participants

Seventy healthy young Chinese adults were recruited from the Shanghai community through online platforms and public advertisements. The inclusion criteria were as follows: (1) age between 18 and 35 years; (2) right-handedness; (3) completion of at least a high school education; (4) normal or corrected-to-normal vision; (5) voluntary participation with signed informed consent. Exclusion criteria included: (1) refusal or inability to provide accurate information; (2) recent use of antibiotics or receipt of any ongoing clinical treatment; (3) diagnosis of psychiatric disorders, neurological diseases, or other primary medical conditions; (4) history of smoking or alcohol abuse; (5) contraindications to MRI scanning, such as metallic implants. All participants provided written informed consent, adhering to the principles outlined in the Declaration of Helsinki. The present study was approved by the Institutional Review Board of Shanghai University of Sports. Participants received financial compensation for their time.

The participants were classified into two groups: a regular physical activity group (RPG) and an inactive group (IAG). The PA level was assessed using the International Physical Activity Questionnaire–Short Form (IPAQ–SF). People who selected “prefer not to answer” or “do not know” were excluded from further analysis. Individuals were categorized as engaging in regular PA if they met all of the following criteria: (1) reporting ≥3 days per week of moderate-or vigorous-intensity PA; (2) a duration of ≥30 min per session; (3) the total PA volume ≥ 600 MET-min/w (metabolic equivalent of task minutes per week) [28]. Furthermore, they had participated in PA at least twice a week for ≥30 min per session during the previous six months [29]. METs values were calculated according to IPAQ scoring guidelines: total minutes per week of moderate-intensity PA were multiplied by 4 METs, and vigorous-intensity PA by 8 METs [30]. In contrast, participants in IAG reported no regular PA during the previous six months and were classified as having the lowest PA level according to IPAQ criteria [30].

### 2.2. Experiment Procedure

All participants completed behavioral assessments and fMRI experiments on the same day. Before the experimental session, they were instructed to refrain from vigorous PA for 24 h and to abstain from caffeinated products for at least 12 h. In addition, participants were required to fast for at least four hours before scanning for standardization. fMRI Scanning sessions were conducted either in the late morning (11:00 a.m.–12:00 p.m.) or in the late afternoon (4:30 p.m.–5:30 p.m.).

On the experimental day, participants first completed a battery of paper-to-pencil questionnaires, including informed consent, the Edinburgh Handedness Inventory, a medical history form, a demographic questionnaire, the IPAQ–SF, and the Beck Depression Inventory–II [31]. Anthropometric measurements, including body mass index (BMI), waist circumference, and visceral fat area, were subsequently measured. Moreover, subjective appetite sensations were assessed immediately before the formal fMRI session using a visual analog scale to control for baseline appetite state. The visual analog scale consisted of four items: (1) “How hungry do you feel right now?”; (2) “How full do you feel right now?”; (3) “How strong is your desire to eat right now?”; (4) “How much food could you eat right now?”. Participants indicated their responses by marking a 100-mm horizontal line anchored by “Not at all” and “Extremely.” A composite appetite score was calculated as follows: (hunger + [100-fullness] + desire to eat + prospective food consumption)/4 [32].

#### 2.2.1. Anthropometric Measurements

Body weight and visceral fat area were assessed using a bioelectrical impedance analyzer (InBody720; InBody Co., Ltd., Cheonan, South Korea) [33]. Body height was measured in duplicate using a stadiometer, and mean values were used for analysis. Waist circumference was measured with participants standing upright, feet together, and arms relaxed at their sides. The measurement site was identified as the midpoint between the lower edge of the rib cage and the iliac crest.

#### 2.2.2. Stop Signal Task

The SST was programmed and administered using E-prime software (version 3.0). Visual stimuli were projected onto a screen positioned behind the participant’s head via an LCD projector (MagnetVision; Resonance Technology Inc., Northridge, CA, USA) and viewed through a mirror mounted on the MRI head coil. Before the formal experiment, participants completed a short practice session to ensure task comprehension. Participants proceeded to the formal task once they achieved approximately 50% stopping accuracy, which is consistent with the requirements of the SST.

The SST was designed to assess IC under two experimental conditions: a food condition, assessing food-specific IC using high-calorie food images, and a neutral condition, assessing general IC using non-food neutral images. The stimuli consisted of 25 high-calorie food images and 25 neutral images, selected from the validated Food-Pics database [34]. Each image was modified to create four visual variants (white background with a red frame, white background with a yellow frame, gray background with a red frame, and gray background with a yellow frame), resulting in 100 unique stimulus configurations per condition [35]. Each configuration was presented twice, resulting in a total of 200 trials per condition.

**Figure 1:**
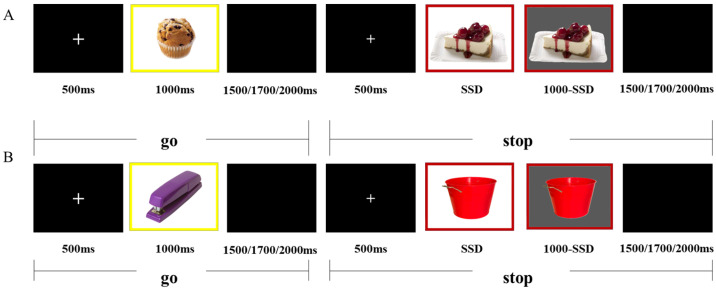
Experimental procedure of the stop-signal task under the high-calorie food condition (**A**) and the neutral condition (**B**). Participants performed go and stop trials while responding to food-related or neutral stimuli during fMRI scanning. The “+” was presented at the center of the screen as a fixation point before each trial. SSD: stop-signal delay.

Each condition comprised 150 go trials (75%) and 50 stop trials (25%) [36]. As illustrated in Figure 1, go trials began with a 500 ms fixation cross, followed by the presentation of a go stimulus for up to 1000 ms. Participants were instructed to respond as quickly and accurately as possible by pressing the “1” key for white backgrounds with red frames and the “3” key for white backgrounds with yellow frames. Stimulus presentation terminated immediately upon response or after the response window elapsed. A jittered inter-trial interval (1500, 1700, or 2000 ms) followed stimulus offset. In stop trials, the go stimulus was followed by a stop signal (gray-background version of the stimulus) after a variable stop-signal delay (SSD). The initial SSD was set at 250 ms and dynamically adjusted using a staircase procedure with 50 ms step increments. Successful inhibition led to an increase in SSD, whereas failed inhibition resulted in a decrease, maintaining an approximate 50% inhibition rate. SSD values were constrained between 50 ms and 1000 ms. Each condition lasted approximately 10 min. The order of the food and neutral sessions was counterbalanced across participants.

#### 2.2.3. Imaging Data Acquisition and Preprocessing

Magnetic resonance imaging data were collected with a 3.0T MAGNETOM Prisma scanner (Siemens Healthcare, Erlangen, Germany) using a 64-channel head-neck coil at Shanghai University of Sport. Participants were positioned supine, provided with hearing protection, and instructed to remain still throughout the scanning procedure.

High-resolution T1-weighted structural images were obtained utilizing a magnetization-prepared rapid gradient echo sequence with the following parameters: repetition time = 2500 ms, echo time = 2.98 ms, inversion time = 1100 ms, 192 slices, slice thickness = 1.0 mm, flip angle = 7°, field of view = 256 × 256 mm^2^, and voxel size = 1.0 × 1.0 × 1.0 mm^3^. Functional images were collected using the echo-planar imaging sequence with the following parameters: repetition time = 2000 ms, echo time = 30 ms, field of view = 220 × 220 mm^2^, 64 slices, slice thickness = 2.5 mm, number of averages = 1, flip angle = 90°, voxel size = 2.5 × 2.5 × 2.5 mm^3^.

Functional imaging data were preprocessed using SPM12 (http://www.fil.ion.ucl.ac.uk/spm/, accessed on 4 November 2018) implemented in MATLAB R2020b. All structural and functional images were converted from DICOM format to NIfTI format. The initial four volumes of each functional run were discarded to allow for magnetic field equilibration. Slice timing correction was performed, followed by head motion realignment. Participants with head motion exceeding 3 mm in translation or 3° in rotation were excluded. Each participant’s T1-weighted image was coregistered to the mean functional image and subsequently segmented into gray matter, white matter, and cerebrospinal fluid using tissue probability maps. The segmented images were spatially normalized to the Montreal Neurological Institute (MNI) 152 standard space using affine linear transformations. The resulting normalization parameters were applied to the functional images, which were resampled to an isotropic voxel size of 3 × 3 × 3 mm^3^. Finally, spatial smoothing was carried out using a Gaussian kernel with a full width at half maximum of 5 mm.

### 2.3. Statistical Analysis

#### 2.3.1. Behavioral Analysis

In the SST, the following behavioral measures were calculated: SSRT, mean SSD, probability of responding on a stop trial [p(respond|signal)], go-trial RT, and go-trial accuracy [26,27]. According to the horse-race model, SSRT serves as a quantitative index of IC efficiency, with shorter SSRTs indicating superior inhibitory performance [26,27].

SSRT was calculated using the integration approach in a customized MATLAB R2020b pipeline as follows: SSRT = RTn − mean (SSD) [24]. The nth RT, representing the finishing time of the stop process, was derived from the go-trial RT distribution. Specifically, go-trial RTs were sorted in ascending order, and the RT at the p(respond|signal) percentile of this ranked distribution was selected as the nth RT. The go trials with no response or with RTs exceeding 1000 ms were replaced with the participant-specific maximum valid go-trial RT. Participants were excluded from behavioral analyses if any of the following criteria were met: (1) mean RT on incorrect stop trials exceeded mean RT on go trials; (2) p(respond|signal) < 25% or >75%; (3) omission rate of go trials exceeded 20% [37].

Group differences in demographic variables (age, BMI, waist circumference, visceral fat area, METs, depressive scores, and composite appetite scores) were assessed using independent-samples *t*-tests. Sex differences were evaluated with chi-square tests. SST behavioral outcomes were analyzed using repeated-measures ANCOVA, with Group (RPG vs. IAG) as the between-subject factor and Stimulus type (high-calorie food vs. neutral images) as the within-subject factor. Age, sex, BMI, depressive scores, and composite appetite scores were included as covariates. All behavioral analyses were conducted with SPSS 29.0. Greenhouse–Geisser corrections were applied when sphericity assumptions were violated, and Bonferroni corrections were used for post hoc comparisons. A significance level of *p* < 0.05 was adopted.

#### 2.3.2. Imaging Data Analysis

For individual-level analysis, a general linear model was employed to construct a multiple regression design matrix that included eight types: food_gocor, food_stopcor, neutral_gocor, neutral_stopcor, food_goincor, food_stopincor, neutral_goincor, neutral_stopincor. Event onsets were time-locked to stimulus presentation and convolved with the canonical hemodynamic response function and its first-order time derivative, with event durations of 0 s. Six head motion parameters obtained from realignment were included as nuisance regressors.

Contrast images representing successful inhibition (stopcor > gocor [38]; stopcor > baseline [39]) and failed inhibition (stopincor > gocor [38,40]) were computed separately for food and neutral conditions for each participant and used in group-level analysis. Notably, the stopincor > gocor contrast involved a motor response in both cases, which helps minimize confounding motor activity with error processing [38,40].

For group-level analyses, a flexible factorial model was constructed with “Group” as the between-subjects factor and “Stimulus type” as the within-subjects factor, controlling for age, sex, BMI, depressive scores, and composite appetite scores. Main effects and interaction effects were tested using F-contrasts. Statistical significance was set at *p* < 0.005 (uncorrected at the voxel level) combined with *p* < 0.05 (family-wise error corrected (FWEcor) at the cluster level), with a minimum cluster size of 20 voxels. Peak coordinates were reported in MNI space.

For region of interest (ROI) analysis, Marsbar (version 0.44) was used to extract mean signal values across all voxels within each ROI. Two types of functional ROI were examined: (1) a priori ROI, defined based on previous literature on IC, specifically the right inferior frontal gyrus [41]; and (2) exploratory ROI, defined based on significant clusters identified in the whole-brain analyses. The right inferior frontal gyrus was defined as an 8 mm radius sphere centered at MNI coordinates (*x* = 51, *y* = 16, *z* = 18), based on a previous meta-analysis [5]. Mean signal values were extracted for the stopcor > gocor and stopcor > baseline contrasts. Pearson correlation analyses were then conducted to examine the associations between SSRT, PA levels (in METs), and ROI activation values. Analyses involving ROIs derived from whole-brain results were considered exploratory and were not used for confirmatory statistical inference.

#### 2.3.3. Sensitivity Analyses

To evaluate the robustness of our primary findings against potential confounding factors, we conducted a series of post hoc sensitivity analyses using four nested statistical models with progressively simplified sets of covariates. The primary model (Model 1) adjusted for age, sex, BMI, depressive scores, and composite appetite scores, representing the main analysis reported above. Model 2 excluded BMI, adjusting for age, sex, depressive scores, and composite appetite scores; Model 3 excluded sex, adjusting for age, BMI, depressive scores, and composite appetite scores; and Model 4 (Minimal Model) included only age, depressive scores, and composite appetite scores as covariates. SSRT and whole-brain analyses were performed across all models to assess the consistency of the results. This approach allowed us to determine whether the observed associations between regular PA and IC were robust to different covariate adjustments, without implying causality.

## 3. Results

### 3.1. Demographic Characteristics

The final sample consisted of sixty-one participants (30 with regular PA and 31 inactive). Four participants were excluded due to excessive head motion (>3 mm translation), two for high accuracy (>75%) on stop trials in the SST, and four for selecting “prefer not to answer” or “do not know” in the IPAQ-SF. The RPG and the IAG were similar in age, waist circumference, composite appetite scores, and depressive scores (see Table 1). Significant differences were found between groups in sex distribution, BMI, visceral fat area, and METs (*p* < 0.05).

### 3.2. Behavioral Performance

**Figure 2:**
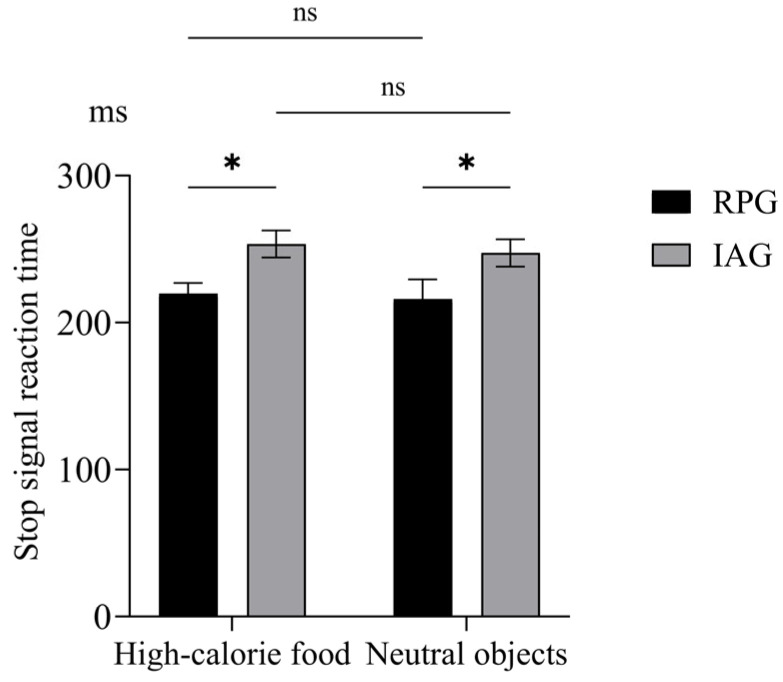
Stop-signal reaction time (SSRT) difference between the two groups under high-calorie food and neutral conditions. RPG, regular physical activity group; IAG, inactive group. * *p* < 0.05. ns, not significant.

Behavioral performance on the SST is summarized in Table 2. After adjusting for covariates (age, sex, BMI, depressive scores, and composite appetite scores), SSRT analysis revealed a significant main effect of group, F (1, 54) = 4.13, *p* = 0.047, *η*^2^ = 0.071. Post hoc analysis showed that the RPG exhibited a shorter SSRT than the IAG (Figure 2). Neither the main effect of stimuli nor the group × stimuli type interaction reached significance. No significant group or stimulus type effects, nor interaction, were observed for SSD, p(respond|signal), or RT of go trials.

### 3.3. Imaging Results

Whole-brain analysis: For the stopcor > gocor contrast, no significant main effects or interactions were observed after controlling for covariates. In contrast, the stopcor > baseline contrast revealed a significant group × stimuli type interaction in a cluster encompassing bilateral precuneus (Table 3, Figure 2). Simple effect analyses showed that under food conditions, the IAG exhibited lower activation than the RPG, whereas under neutral conditions, the RPG showed lower activation than the IAG. Within-group comparisons revealed that neutral stimuli elicited lower activation than food stimuli in the RPG, whereas food stimuli elicited lower activation in the IAG.

**Figure 3:**
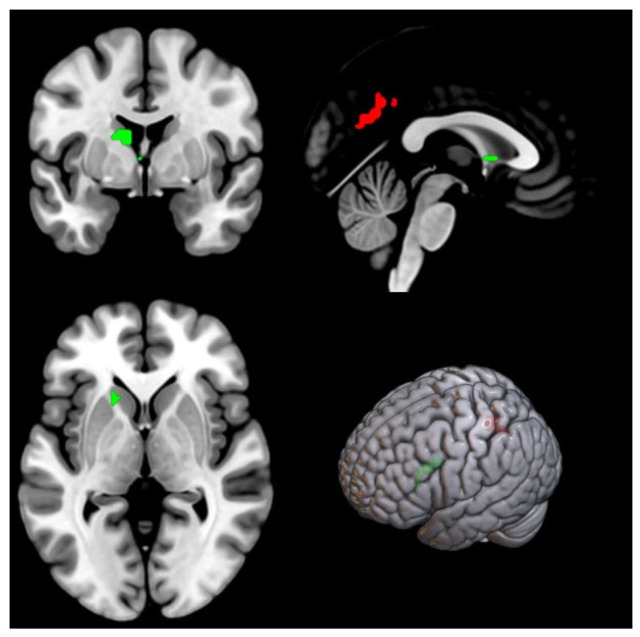
Brain regions showing significant signal differences in the bilateral precuneus (red) and left caudate (green) during the stop-signal task.

For the stopincor > gocor contrast, a significant group × stimuli type interaction was observed in the left caudate (Table 3, Figure 3). In the food condition, the IAG exhibited higher activation than the RPG. Within-group comparisons showed that in the RPG, food stimuli elicited lower activation than neutral stimuli, whereas the opposite pattern was observed in the IAG.

**Figure 4:**
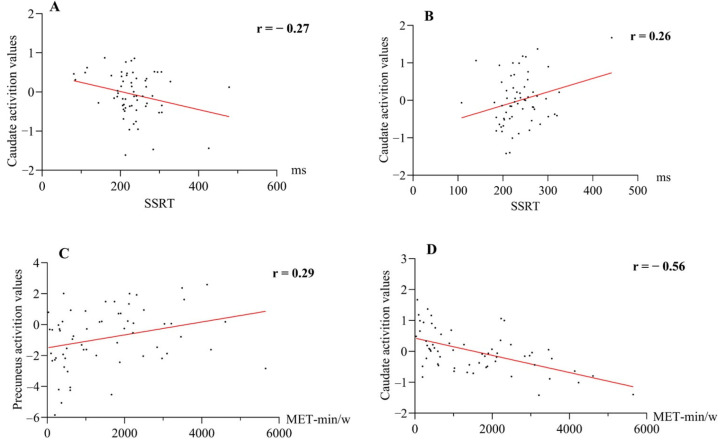
Associations between region-of-interest (ROI) BOLD signal values, stop-signal reaction time (SSRT), and METs in healthy adults. (**A**) Correlation between left caudate signal values and SSRT under the neutral condition. (**B**) Correlation between left caudate signal values and SSRT under the high-calorie food condition. (**C**) Correlation between METs and bilateral precuneus activation under the high-calorie food condition. (**D**) Correlation between METs and left caudate activation under the high-calorie food condition. All correlations are exploratory and were not used for confirmatory statistical inference. The red line represents the linear fit from the Pearson correlation analysis.

Functional ROI analysis: For the right inferior frontal gyrus, no significant correlations were observed between mean signal values and either SSRT or METs in either stimulus condition (all *p* > 0.05). In exploratory ROI analysis, stimulus-dependent associations were observed in the left caudate and precuneus. Under the neutral condition, SSRT was negatively correlated with left caudate activity (*r* = −0.267), whereas under the food condition, SSRT was positively correlated with left caudate activity (*r* = 0.263). In addition, METs were positively associated with bilateral precuneus activation (*r* = 0.29) and negatively related to left caudate activation (*r* = −0.56) under the food condition. These exploratory associations are illustrated in Figure 4.

### 3.4. Results of Sensitivity

To assess the potential confounding effects of sex and BMI, additional Post hoc sensitivity analyses were conducted. Detailed results for SSRT and brain activation across four models are presented in Supplementary Table S1. For bilateral precuneus activation during successful inhibition (stopcor > baseline), the group × stimulus type interaction was significant in Models 1 and 2, while in Models 3 and 4, the effect did not reach significance but showed a similar trend. Left caudate activation during failed inhibition (stopincor > gocor) remained significant across all models. The main group effect (RPG < IAG) on SSRT was consistent across all models. Overall, these findings indicate that the observed behavioral and neural differences are generally robust to covariate adjustments, although significance levels varied slightly depending on the model.

## 4. Discussion

This study investigated the behavioral and neural correlates of IC during exposure to high-calorie food cues in individuals with regular PA compared to inactive individuals. At the behavioral level, RPG exhibited significantly shorter SSRT than IAG across both neutral and food conditions, consistent with prior studies showing that higher levels of PA enhance general IC in healthy populations [7,24,29,42,43]. Importantly, the present findings extend this literature by demonstrating that this association is also evident in high-calorie food-related inhibitory contexts, which are particularly relevant in obesogenic environments. At the neural level, regular PA was associated with stimulus-dependent neural activity patterns in bilateral precuneus and left caudate, suggesting that PA relates not only to behavioral performance but also to underlying neural processes supporting IC under motivationally salient conditions.

During the successful inhibition (stopcor > baseline), the RPG exhibited lower activation in bilateral precuneus than IAG under neutral conditions, whereas IAG displayed lower activation than RPG under high-calorie food conditions. The precuneus, as a core hub of the posterior DMN, is implicated in internally oriented cognitive processes [44,45,46]. Reduced activation in this region has been associated with a shift from internally directed cognition to externally oriented executive control [47]. In this context, lower precuneus activation in the RPG under neural conditions may be related to more efficient suppression of task-irrelevant internal processes during inhibitory demands. This interpretation aligns with the effort hypothesis, which posits that repeated engagement in cognitively demanding PA could enhance the capacity to regulate neural resources when focused attention is required [1]. Meta-analytic [25] and intervention [23,24] studies further reported that PA could modulate neural activity within the DMN, including the precuneus.

Conversely, the pattern observed in the IAG under high-calorie food conditions may reflect a different cognitive context. High-calorie food stimuli are inherently salient and motivationally relevant, engaging both reward-related and attentional systems [48]. Successfully inhibiting responses in the presence of such cues could therefore require additional cognitive effort. Accumulating evidence suggests that the brain regions involved in internal processes dynamically interact with cognitive control networks in a manner that depends on cognitive effort [6,49,50]. From this perspective, reduced precuneus activation in IAG under high-calorie food conditions may be related to the increased cognitive effort required to counteract the salience of food cues and maintain IC. The positive association between PA levels and precuneus activation during food-related inhibition further suggests that regular PA is linked to stimulus-dependent modulation of bilateral precuneus engagement. However, the contrast for stopcor > baseline was more closely linked to the global process of implementing successful inhibition within a task context, rather than to a specific mechanism of action cancellation. Future studies with larger samples are needed to disentangle these global effects from more specific components of IC by directly comparing different analytical contrasts.

Additional insights emerged from failed inhibition trials, which revealed stimulus-dependent modulation of the left caudate. In inactive individuals, high-calorie food cues elicited more pronounced caudate responses compared with neutral stimuli, whereas chronic active individuals showed the opposite pattern. Moreover, greater caudate activation under food conditions was associated with longer SSRT and lower PA levels. The caudate nucleus was critically involved in reward processing and motivational salience [51,52], and exaggerated striatal responses to palatable food cues have been linked to impulsive eating behaviors and increased obesity risk [7]. The present findings suggest that heightened caudate neural responses may interfere with the process of success inhibition when food salience is high, particularly among inactive individuals. Although these associations are correlational and should be interpreted cautiously, they are consistent with prior neuroimaging studies reporting attenuated food-related reward responses in key reward regions, including the orbitofrontal cortex, putamen, and amygdala, among individuals with higher PA levels [14,15,53]. The present task-based results suggest that PA–related differences in reward responsivity could become especially evident in situations requiring active self-regulation.

It is important to emphasize that the present findings are associative. The neurocognitive advantages observed in the RPG may not stem totally from the physiological effects of PA per se, but could partly reflect the ability of self-control, which may be influenced by genetics and environment [1,54]. The virtuous circle model further suggests the bidirectional relationship between PA and cognition. On the one hand, chronic regular PA benefits cognitive control and brain regions involved in effort-based decision making or self-control; on the other hand, IC, self-control, and effortful control could enhance adherence to PA. Longitudinal or intervention studies that specifically measure such psychological constructs are necessary to disentangle the unique contribution of PA from the potential effects of this shared underlying mindset.

Furthermore, group differences in BMI and sex distribution could also have contributed to the observed behavioral performance and neural activity differences. Previous studies have reported that higher BMI was associated with poorer IC [55,56]. Although regular PA does not necessarily lead to substantial weight loss, adiposity-related outcomes are influenced by multiple factors such as age, exercise prescription, genetic background, environmental context, and metabolic profiles [10]. Importantly, higher levels of PA have been associated with more favorable adiposity profiles and attenuation of weight gain in adults [57]. It has been raised that chronic PA may partially mitigate the negative impact of obesity on cognitive control. A cross-sectional study reported that only obese individuals with sedentary lifestyles, but not those engaging in regular exercise, exhibited prolonged RT during cognitive control tasks [29]. Consistent with this interpretation, when BMI was included as a covariate in the present analyses, the difference in SSRT and brain activation remained robust, indicating that the observed behavioral advantage in the RPG cannot be fully explained by BMI differences alone. Sex-related differences in neural responses during response inhibition have also been reported in prior research [58,59]. However, existing evidence suggests that such differences are modest and highly task-dependent, with within-sex variability often exceeding between-sex variability, including effects related to menstrual cycle phase [60]. In the present study, sex was included as a covariate, and sensitivity analyses indicated that the main findings remained robust after adjustment. Nevertheless, future studies with larger samples are needed to allow stratified analyses across BMI categories and sex distribution.

Several limitations should be acknowledged. First, the cross-sectional design precludes causal inference; longitudinal exercise intervention studies are needed to establish directionality. Second, the relatively small sample size limited the ability to conduct stratified analyses by sex or BMI. Third, PA type was heterogeneous and assessed via self-report rather than objective measures, which may introduce measurement error [12,61]. Fourth, the reliance on the stopcor > baseline contrast, which captures broad task-engagement signals, warrants caution when attributing effects solely to IC. Future studies with more homogeneous samples, objective PA assessment (e.g., accelerometry), and stratified analyses are needed to confirm and extend these findings.

## 5. Conclusions

Regular PA was associated with enhanced IC performance across both neutral and high-calorie food cue conditions, and with stimulus-dependent neural activity patterns in the bilateral precuneus and left caudate. These findings highlight the potential role of PA in supporting IC when individuals are confronted with highly salient food cues in obesogenic environments. Given the relatively small sample size and the unbalanced distribution of sex and BMI, replication in larger, more representative samples using objective measures of PA is warranted.

## Figures and Tables

**Table 1:** Demographic and anthropometric characteristics of participants in the regular physical activity group (RPG) and the inactive group (IAG).

	RPG (N = 30)	IAG (N = 31)	*p*	Post Hoc
Sex (male/female)	24/6	17/14	*p* = 0.036 *	Male: RPG > IAG Female: RPG < IAG
Age (years)	21.30 ± 3.51	20.65 ± 2.24	*p* = 0.388	—
BMI (kg/m^2^)	27.36 ± 5.36	30.69 ± 6.09	*p* = 0.027 *	RPG < IAG
Visceral fat area (cm^2^)	99.62 ± 52.06	134.23 ± 49.27	*p* = 0.010 *	RPG < IAG
Waist (cm)	88.51 ± 12.44	94.81 ± 12.98	*p* = 0.058	—
Composite appetite scores	57.00 ± 16.42	57.86 ± 18.88	*p* = 0.850	—
BDI-II scores	7.47 ± 5.35	8.87 ± 5.73	*p* = 0.327	—
IPAQ-SF scores (METs)	2501.65 ± 1130.04	631 ± 713.06	*p* < 0.001 **	RPG > IAG

Values are presented as mean ± SD. * *p* < 0.05; ** *p* < 0.005. BDI-II, Beck Depression Inventory-II; BMI, body mass index; IPAQ-SF, International Physical Activity Questionnaire-Short Form. METs, metabolic equivalent.

**Table 2:** Behavioral performance on the stop-signal task (SST) in the regular physical activity group (RPG) and the inactive group (IAG).

Conditions	Variables	RPG (N = 30)	IAG (N = 31)
High-calorie food stimuli	SSRT	219.77 ± 39.57	253.45 ± 51.40
	SSD	292.20 ± 72.06	277.97 ± 99.42
	p(respond|signal)	0.49 ± 0.03	0.50 ± 0.03
	Accuracy of go trials	0.93 ± 0.04	0.92 ± 0.04
	RT of go trials	521.44 ± 62.73	538.04 ± 83.96
Neutral stimuli	SSRT	215.97 ± 73.33	247.45 ± 51.93
	SSD	288.52 ± 82.75	281.52 ± 100.85
	p(respond|signal)	0.50 ± 0.08	0.50 ± 0.03
	Accuracy of go trials	0.92 ± 0.04	0.92 ± 0.06
	RT of go trials	514.74 ± 65.02	535.71 ± 85.87

SSRT, stop-signal reaction time; SSD, stop-signal delay; p(respond|signal), probability of responding given a stop signal (inhibition failure rate); RT, reaction time. SSRT values were calculated separately for food and neutral conditions.

**Table 3:** Whole-brain activation differences between the regular physical activity group (RPG) and the inactive group (IAG) during the stop-signal task.

Contrast	MNI Coordinate			P_FWE-corr_	Z	Voxel	Regions of Activation
	*X*	*Y*	*Z*				
Stopcor > baseline	6	−54	36	0.036	3.59	65	
						33	Precuneus_R
						20	Precuneus_L
						5	Cingulate_Post_R
						3	Cuneus_L
						3	Cingulate_Mid_R
						1	Cingulate_Mid_L
Stopincor > gocor	−15	9	12	0.040	3.94	63	
						42	Caudate_L
						3	Putamen_L
						2	Thal_VA_L

## Data Availability

The original contributions presented in this study are included in the article/Supplementary Material. Further inquiries can be directed to the corresponding author.

## Supplementary Materials

The following supporting information can be downloaded at: https://www.mdpi.com/article/10.3390/nu18030486/s1, Table S1: Sensitivity analyses of stop-signal reaction time and whole-brain level activation across four models.

## Abbreviations

The following abbreviations are used in this manuscript:

BMI	Body mass index
DMN	Default mode network
fMRI	Functional magnetic resonance imaging
FWEcorr	Family-wise error corrected
IAG	Inactive group
IC	Inhibitory control
IPAQ–SF	The International Physical Activity Questionnaire–short form
MET	Metabolic equivalent of task
MNI	Montreal Neurological Institute
PA	Physical activity
p(respond|signal)	Probability of responding on a stop trial
ROI	Region of Interest
RPG	Regular physical activity group
RT	Reaction time
SSD	Stop-signal delay
SSRT	Stop-signal reaction time
SST	Stop signal task
