# Supplementary material for: Association Between Regular Physical Activity and Food-Specific Inhibitory Control in Young Chinese Adults: An fMRI Study
Source: Nutrients. 2026 Feb 1;18(3):486. doi: 10.3390/nu18030486 (PMC12899968; doi:10.3390/nu18030486)
Supplement: Supplementary file 1 [file nutrients-18-00486-s001.zip › nutrients-4111621-supplementary.pdf]

Supplementary material

Table S1 Sensitivity analyses of stop-signal reaction time and whole-brain level activation across four models.

Model	Contrast	MNI coordinate			$P_{FWE\text{-}corr}$	Z	Voxel	Mian Regions of activation	SSRT
		X	Y	Z					
Model 1	Adjusted for Age + BDI + composite appetite score +Sex + BMI								
	Stopcor > baseline	6	-54	36	0.036	3.59	65	Bilateral Precuneus	Group main effect significant, p=0.047, RPG < IAG
	Stopincor > gocor	-15	9	12	0.040	3.94	63	Caudate_L	
Model 2	Adjusted for Age + BDI + composite appetite score +Sex								
	Stopcor > baseline	6	-54	36	0.048	3.59	61	Bilateral Precuneus	Group main effect significant, p=0.013, RPG < IAG
	Stopincor > gocor	-15	9	12	0.041	3.96	63	Caudate_L	
Model 3	Adjusted for Age + BDI + composite appetite score +BMI								
	Stopcor > baseline	6	-54	36	0.051	3.60	61	Bilateral Precuneus	Group main effect significant, p=0.046, RPG < IAG
	Stopincor > gocor	-15	9	12	0.040	3.96	63	Caudate_L	
Model 4	Adjusted for Age + BDI +composite appetite score								
	Stopcor > baseline	6	-54	36	0.069	3.59	57	Bilateral Precuneus	Group main effect significant, p=0.012, RPG < IAG
	Stopincor > gocor	-15	9	12	0.038	3.97	64	Caudate_L	

Note: BDI, Beck Depression Inventory–II; BMI, Body Mass Index.
